# Correction: Preparation of gypsum/sawdust green composite with spray coating

**DOI:** 10.1039/d3ra90007j

**Published:** 2023-01-19

**Authors:** Dasong Dai, Mizi Fan

**Affiliations:** a College of Material Engineering, Fujian Agriculture and Forestry University Fuzhou 350002 PR China; b Nanocellulose and Biocomposites Research Centre, College of Engineering, Design and Physical Sciences, Brunel University UB8 3PH UK mizi.fan@brunel.ac.uk

## Abstract

Correction for ‘Preparation of gypsum/sawdust green composite with spray coating’ by Dasong Dai *et al.*, *RSC Adv.*, 2015, **5**, 96965–96971, https://doi.org/10.1039/C5RA18707A.

The authors wish to draw the readers’ attention to their closely related paper, in *Ind. Crop & Prod. A*,^1^ which should have been cited in this *RSC Advances* paper.

The authors regret the unattributed text, figure and data overlap between their article and ref. 1. Table 1, Fig. 2, Fig. 3 and Table 2 were re-used in part or in full from ref. 1 without being correctly attributed and without permission from the Publisher.

The authors have now received the permission to reuse the images and the corrected captions are shown below:

Table 1 Effect of SW water extractives on the mechanical performance of gypsum. Reprinted in part from Dasong Dai and Mizi Fan, ‘Preparation of bio-composite from wood sawdust and gypsum’, *Ind. Crops Prod.*, 2015, **74**, 417–424, with permission from Elsevier.

Fig. 2 ATR-FTIR spectra of WBE coating SW, raw SW and subtraction from the surface of WBE coating SW. Reprinted from Dasong Dai and Mizi Fan, ‘Preparation of bio-composite from wood sawdust and gypsum’, *Ind. Crops Prod.*, 2015, **74**, 417–424, with permission from Elsevier.

Fig. 3 Matching result of subtraction from the surface of WBE coating sawdust with OMNIC software. Reprinted from Dasong Dai and Mizi Fan, ‘Preparation of bio-composite from wood sawdust and gypsum’, *Ind. Crops Prod.*, 2015, **74**, 417–424, with permission from Elsevier.

Table 2 Effect of SW modification on the mechanical performance of gypsum/SW composite (with ultrasonic pretreatment). Reprinted in part from Dasong Dai and Mizi Fan, ‘Preparation of bio-composite from wood sawdust and gypsum’, *Ind. Crops Prod.*, 2015, **74**, 417–424, with permission from Elsevier.

The Royal Society of Chemistry apologises for these errors and any consequent inconvenience to authors and readers.

## Supplementary Material
